# Can Brazil play a more important role in global tuberculosis drug production? An assessment of current capacity and challenges

**DOI:** 10.1186/1471-2458-13-279

**Published:** 2013-03-27

**Authors:** Andre Gemal, Joel Keravec, Alexandre Menezes, Anete Trajman

**Affiliations:** 1 Federal University of Rio de Janeiro and National Institute of Health Quality Control/Fiocruz, Rio de Janeiro, Brazil; 2 Management Sciences for Health, Projeto MSH, Rio de Janeiro, Brazil; 3 Global Health Strategies, Rio de Janeiro, Brazil; 4 Gama Filho University, Rio de Janeiro, Brazil; 5 McGill University, Montreal, Canada; 6 Universidade Gama Filho, Mestrado Profissional em Ensino na Saúde, Rua Manoel Vitorino 553, Prédio AG, 5o andar, Rio de Janeiro, Piedade, 20748-900, Brazil

**Keywords:** Antitubercular agents, Certification, Fixed dose combination, Pharmaceuticals, Quality control, Tuberculosis

## Abstract

**Background:**

Despite the existence of effective treatment, tuberculosis is still a global public health issue. The World Health Organization recommends a six-month four-drug regimen in fixed-dose combination formulation to treat drug sensitive tuberculosis, and long course regimens with several second-line drugs to treat multi-drug resistant tuberculosis. To achieve the projected tuberculosis elimination goal by 2050, it will be essential to ensure a non-interrupted supply of quality-assured tuberculosis drugs. However, quality and affordable tuberculosis drug supply is still a significant challenge for National Tuberculosis Programs.

**Discussion:**

Quality drug production requires a combination of complex steps. The first challenge is to guarantee the quality of tuberculosis active pharmaceutical ingredients, then ensure an adequate manufacturing process, according to international standards, to guarantee final product´s safety, efficacy and quality. Good practices for storage, transport, distribution and quality control procedures must follow. In contrast to other high-burden countries, Brazil produces tuberculosis drugs through a strong network of public sector drug manufacturers regulated by a World Health Organization-certified national sanitary authority. The installed capacity for production surpasses the 71,000 needed treatments in the country. However, in order to be prepared to act as a global supplier, important bottlenecks are to be overcome. This article presents an in-depth analysis of the current status of production of tuberculosis drugs in Brazil and the bottlenecks and opportunities for the country to sustain national demand and play a role as a potential global supplier. Raw material and drug production, quality control, international certification and pre-qualification, political commitment and regulatory aspects are discussed, as well recommendations for tackling these bottlenecks. This discussion becomes more important as new drugs and regimens to treat tuberculosis are expected in a close future.

**Summary:**

International manufacturers of raw material for tuberculosis treatment should undergo certification and pre-qualify their active pharmaceutical ingredients as a first step to ensure quality of tuberculosis drugs. At the country level, Brazilian public manufacturers should apply for international certification and tuberculosis drugs should be pre-qualified by international organisms. Finally, only with political commitment and large-scale production will Brazilian public sector manufacturers be able to partially supply the global market.

## Background

The World Health Organization (WHO) estimates that one third of the world population is infected by *M. tuberculosis*. WHO also estimates that in 2010, 8.7 million (range 8.3-9.0 million) people developed tuberculosis (TB) and 0.99 million (range 0.84-1.1 million) died from TB, with 0.43 million (range 0.40-0.46 million) additional deaths from HIV-associated TB. [1] Worldwide, TB is the second leading cause of death from infectious diseases [1] and the first among people living with HIV/Aids. [2] To combat TB worldwide, the United Nations [3] and WHO [4] propose that by 2050, TB global incidence rate should decrease to less than 1/1,000,000 inhabitants per year. By 2015, global targets aim to reduce global TB prevalence and death rates by 50% compared to 1990 [3].

Among other challenges, the rapid spread of drug resistant TB in Africa, Eastern Europe and Asia [5,6] jeopardizes the achievement of these goals [1]. Drug resistant strains emerged mainly from the inadequate use of TB drugs, or use of low quality drugs, poor TB program performance and lack of regulation [7]. The incorporation at country level of promising advances on new drug, vaccine and diagnostic technologies will still take time before they can effectively contribute to global TB control [8,9]. In the meantime, it is important to guarantee an uninterrupted supply of quality-assured drugs at the country level.

This article will introduce key issues on the Brazilian model for TB control and focus on the production of quality TB drugs in Brazil, highlighting current strengths and obstacles of the Brazilian Public Pharmaceutical Manufacturing Laboratories (PPML) [10] as potential suppliers for the global market.

## Discussion

### Specificities of Brazilian Context for TB Control

Brazil is one of the 22 countries that account for 80% of the global burden of TB [1]. In 2010, the country reported 71,337 new TB cases [1]. In Brazil, TB treatment is offered exclusively in the public sector, according to current rules and treatment protocols recommended by the Ministry of Health (MoH) [11]. Public manufacturers provide TB drugs, including fixed-dose combination (FDC) of rifampicin and isoniazid since the 1970s, [9] which are distributed free of charge across the country [12]. The drugs are purchased centrally by the MoH and distributed to the municipalities [13]. Except for quinolones and aminoglycosides, TB drugs are not available in private pharmacies; they are exclusively distributed in the public health system. Treatment of TB is based on national recommendations and guidelines edited jointly by the MoH and professional associations [11,14]. There is no private market for TB drugs or TB treatment. As a consequence, the rate of multidrug-resistant TB in the country has remained low (1.4%, personal communication by Brazilian National TB Control Program (NTP), based on national survey conducted in 2008–2009).

### Opportunities for TB drug manufacturing in Brazil

Drug production is regulated by Brazil’s National Regulation Authority (ANVISA) [15]. ANVISA was established in 1999 to regulate health products and services in Brazil, following the model of international regulation authorities,[15] and is WHO pre-qualified for regulation of vaccine production [15]. The PPML produce 11 billion pharmaceutical units per year to meet the needs of government-run public health programs [10,16]. This manufacturing network has improved technologically and gained recognition through its broadly acknowledged capacity for antiretroviral production [13,17]. First-line TB drugs, including fixed dose combination formulations, and some second-line TB drugs are currently manufactured by these PPML.[17] Farmanguinhos, one of the most innovative manufacturers of this network, developed the Rifampicin/Isoniazid (RH) 2:1 FDC tablet (currently undergoing registration process) and is working on the pharmacotechnical development of the Rifampicin/Isoniazid/Pyrazinamide/Ethambutol (RHZE) 4:1 FDC, through a public-private partnership (PPP) agreement with a WHO-pre-qualified Indian manufacturer [18].

Under this new context, PPML produce TB drugs with formulations and dosages/strengths aligned with WHO recommendations, opening new opportunities to supply the international market [17]. Entering the global supply chain would likely contribute to leverage production scale, and maintenance of quality standards without significant extra costs. However, in order to reach international certification requirements, like compliance to WHO’s pre-qualification program, [19] and attain financial sustainability, a few bottlenecks should be overcome.

### Bottlenecks for TB drug manufacturing in Brazil

#### Economic incentives for production

The costs of drugs and the impact of imported supplies on the national trade balance are important issues for the sustainability of any public health system, often influencing priorities for investment. However, the majority of TB drugs are available at low cost in national and international markets [10,20] and therefore have limited budgetary impact for the Brazilian National Health System (*Sistema Único de Saúde -* SUS) [21]. Thus, most TB drugs have low commercial interest for manufacturers, [10,20] including PPML. More expensive second-line drugs, such as capreomycin, cycloserin/terezidon, 4-aminosalicylic acid (PAS) or ultimate generation quinolones, at least while they are not part of first-line regimen, are also not considered a priority for local production, because of Brazil’s low drug resistance rates and limited demand. [1,5] Instead, industrial interests are focused on the most innovative technological approaches to maximize return on investments. The same logic applies to the public sector based industry, which frequently chooses to produce, promote and fund innovation focusing on high-cost drugs that can boost revenues for the PPML, and reduce costs for the health system [10,22]. TB drugs do not represent such an incentive [21,23].

Nevertheless, TB is a national and global priority [10]. It is critical to engage diverse stakeholders, including regulatory agencies, media, civil society and political organizations to support domestic production of TB drugs in Brazil. Even if not profitable, domestic production would benefit job creation, technological development and supply security, and above all, meet an important public health need.

#### Active pharmaceutical ingredients (API)

Manufacturing of most molecules employed in TB treatment does not impose significant technological challenges [24]. In addition to international API manufacturers directly linked to large pharmaceutical companies, countless independent medium-sized companies manufacture API. However, challenges remain, including an economic disincentive (due to low cost and low demand) and gaps in the parameters and mechanisms to guarantee API quality standards [25]. Limited official control from national and international regulatory agencies also leads to highly variable quality of API [1,19,23]. This severely impacts the quality of final products and production consistency.

In order to guarantee drug quality, physicochemical and microbiological characteristics of API must be standardized and kept constant, [24] through an expanded certification process. Certification should be based on specific description of quality parameters for API, and pre-qualification of raw materials is an essential step for the manufacturing of quality drugs [25,26]. Private industries ensure compliance and consistency by establishing supplier-manufacturer agreements. However, Brazilian PPML must follow public sector procurement rules, [27] and government regulations prohibit procurement notices conditioned to specific technical requirements. This has occasionally made it difficult for PPML to purchase critical pharmaceutical ingredients in accordance with characteristics approved by the health regulatory agencies, or to establish a consistent relationship with a selected supplier. These legal procurement aspects, as they apply to API, may hamper the uniformity of nationally manufactured drugs.

### The manufacturing process

Drug quality control, whether carried out by the private industry or by the national regulatory inspection framework, involves a set of legal, technical and administrative requirements [24,28]. In Brazil, quality control is insured by the Brazilian Sanitary Surveillance System, headed by the Brazilian National Institute of Metrology [29] and ANVISA [15]. One potential limiting factor of this highly regulated sector is the availability of reference materials for bioavailability and bioequivalence studies in the national market [30]. In addition, standardized proficiency testing programs must be carried out on a regular basis. Although the government implemented a dedicated quality control program for TB drugs in 2005, [31] and reference substances were prioritized for local development by the Brazilian Pharmacopeia, [30] strong political will and support are necessary to ensure the continuation of such a program. Additionally, it is critical to ensure effective mechanisms for drug certification [25].

### Internal technological development

Over the last few decades, Brazil has increased its investment in scientific and technological innovation. Numerous examples can be found in other fields of knowledge, such as the country’s expanded participation in the petroleum/biodiesel and agricultural global markets [32]. This investment has also led to growth in scientific publications around health, including scholarships and research grants for TB projects [33].

In the past decade, Brazil identified the pharmaceutical sector as a priority for industrial policy [34]. PPPs supporting technology transfers from international companies to domestic public sector manufacturers are a core strategy of that policy. The consolidated experience of the numerous PPPs coupled with national investment in development of new technologies by Brazilian investigators represent an important push for pharmaceutical innovation in the country.

Despite these recent efforts, significant investment is still needed. In the 1990s, rapid changes in importation policies led to a lack of prioritization of industrial drug manufacturing capacity, which had been strengthened under previous national development cycles [34]. This created a gap in pharmaceutical research and development capabilities that is still felt today. Going forward, progress will require strong science and technology policies that encourage later-stage pharmaceutical-technical development and industrial-scale drug manufacturing.

### Recommendations for addressing the identified bottlenecks

#### Economic incentives for production

The SUS strategic product policy could incorporate some of the innovative thinking developed through national and international initiatives such as the Drugs for Neglected Disease Initiative program [35]. To incentivize drug development, this program successfully balanced economic interests and public health needs, including market/supply forecasts, safety and quality-related issues regarding these drugs.

Brazil has been playing an important role in South-South cooperation on health and other development sectors [36]. This has enhanced the country’s political credibility worldwide, and could facilitate access to global markets for domestically-produced TB drugs. Since national demand for TB drugs is relatively limited in scale, participating in international markets through global initiatives would help justify required investments, benefiting manufacturing capacity overall. Additionally, product development efforts focusing on needed innovations for TB control, such as paediatric, geriatric as well as parenteral formulations, may further expand the potential international market for PPML.

Finally, if PPML are fully compliant with international requirements for drug quality, innovative models guaranteeing advanced purchase commitments from international mechanisms would facilitate Brazil engagement for investing in TB drug production.

#### API

The legal requirements regarding the bidding process for API in Brazil need to be revisited to address the PPML specificities and to incorporate a new legal paradigm to increase efficiency for public sector companies. This will require strong political advocacy and commitment, along with improved harmonization across government agencies to further define and adapt legal mechanisms and administrative processes to leverage suitable levels of efficiency in API purchasing. In addition, it will be necessary to define requirements for API certification by national or international organizations. TB API suppliers could be encouraged to register their quality, safety and efficiency standards with national regulatory authorities. Issues to be considered as part of the registration process should include detailed information on the different synthetic routes, specific and significant toxicological impurities, polymorphism among other physicochemical characteristics, which would allow for comparison between API from diverse manufacturers.

This process would allow Brazil and other manufacturing countries to share key updated information on API suppliers. Above all, this approach would enable the Brazilian MoH to monitor API market dynamics so that in critical situations, such as when there are limited manufacturers or competition for specific API, risks of shortages would be minimized and overall API quality standards improved. If WHO and other international organizations standardize and expand their pre-qualification mechanisms, Brazil – and other interested countries - should take part in the process.

If coordinated with WHO and other international organizations, this registration process would likely increase API supplier interest in applying for health registration.

#### The manufacturing process

The Brazilian government should encourage PPML to apply for the WHO pre-qualification program [26] and initiate first and second-line drug regulatory registration procedures in other countries. This could be leveraged through a more unified global strategy approach. In partnership with key international stakeholders and donors, the Brazilian government could develop a priority agenda for global and regional production of TB drugs. In a short time span, Brazil could play an important role in supplying TB drugs to the international market, particularly given the organizational strengths of SUS [13] and the fact that standards for good manufacturing practices (GMP) certification are aligned with the international ones. Farmanguinhos, one of the main PPML, is already GMP certified for some of its products. So is the Navy Forces Laboratory (*laboratório da Marinha*), a fluoroquinolone producer. This needs to be expanded, monitored and encouraged so that it becomes a model for drug development programs at other PPML.

#### Internal technological development

If Brazil wishes to play a significant role as a global TB drug producer, it is essential to continue with the PPP approach for technology transfer and take additional measures to foster domestic investment on pharmaceutical research and innovation.

Incentivizing more interaction between public sector research institutes and pharmaceutical manufacturers through the Brazilian TB Research Network [37] may be an adequate approach to establish innovative partnerships. Moreover, lessons learned during the implementation of a quality-control program for TB drugs in Brazil indicated that there is strong interest in more interactions between the manufacturing sector and the national regulation authority. A closer collaboration between manufacturers and ANVISA would help address, as early as possible, manufacturing challenges that may impact quality of final products. If implemented, these measures may provide the basis for later stage development processes, in case new molecules currently under development are launched as new drugs [38] and attract the attention of the manufacturing sector.

## Summary

Considering the technical capacity, regulation framework and industrial network established by PPML, Brazil has a strong potential for supplying TB drugs to the international market in the near future. However, several issues and bottlenecks still need to be addressed. At the global level, an important step is to ensure the availability of quality API. Brazilian manufacturers should be allowed to purchase of API exclusively from pre-qualified manufacturers, which will require new mechanisms for API certification and procurement by the Brazilian agencies and public administration. Furthermore, the Brazilian public laboratory network needs to seek broader recognition by pursuing certification through international quality mechanisms like the WHO pre-qualification program. TB drug production for international markets could also be included in Brazil’s South-South cooperation agenda. In addition to benefiting global access, these efforts would provide synergistic effects to consolidate capacity for regular quality-assured TB drug production for Brazil’s own domestic demand.

## Abbreviations

4:1: Drug containing four active ingredients (RHZE) in one unit, the tablet; ANVISA: National Health Surveillance Agency; API: Active Pharmaceuticals Ingredients; FDC: Fixed-dose combination; PPML: Public Pharmaceutical Manufacturing Laboratories; MoH: Ministry of Health; NTP: National Tuberculosis Control Program; PPP: Public-Private Partnership; RH: Rifampicin/Isoniazid; RHZE: Rifampicin/Isoniazid/Pyrazinamide/Ethambutol; SUS: Brazilian Unified National Health System; TB: Tuberculosis; WHO: World Health Organization.

## Competing interests

The authors declare no competing interests. The sponsors (Fundação Ataulpho de Paiva, through a grant by Bill and Melinda Gates Foundation) are not responsible for any statements in this manuscript.

## Authors’ contributions

AG interviewed MoH partners, public laboratory leaders, international and non-governmental organizations. All authors discussed the contents of interviews and the recommendations included in the manuscript. AT and AM edited the manuscript. All authors approved the final version of the present manuscript.

## Pre-publication history

The pre-publication history for this paper can be accessed here:

http://www.biomedcentral.com/1471-2458/13/279/prepub
